# Comparable radiation sensitivity in p53 wild-type and p53 deficient tumor cells associated with different cell death modalities

**DOI:** 10.1038/s41420-021-00570-5

**Published:** 2021-07-20

**Authors:** Ping Li, Xiongxiong Liu, Ting Zhao, Feifei Li, Qiqi Wang, Pengcheng Zhang, Ryoichi Hirayama, Weiqiang Chen, Xiaodong Jin, Xiaogang Zheng, Zhen Wang, Qiang Li

**Affiliations:** 1 Institute of Modern Physics, Chinese Academy of Sciences, Lanzhou, China; 2 Key Laboratory of Heavy Ion Radiation Biology and Medicine, Chinese Academy of Sciences, Lanzhou, China; 3 Key Laboratory of Basic Research on Heavy Ion Radiation Application in Medicine, Lanzhou, Gansu Province China; 4 University of Chinese Academy of Sciences, Beijing, China; 5 College of Life Science, Northwest Normal University, Lanzhou, China; 6 Department of Charged Particle Therapy Research, Institute for Quantum Medical Science (iQMS), National Institutes for Quantum and Radiological Science and Technology (QST), Chiba, Japan; 7 Institute of Medicinal Biotechnology, Peking Union Medical College, Chinese Academy of Medical Sciences, Beijing, China

**Keywords:** Apoptosis, Cancer prevention

## Abstract

Studies of radiation interaction with tumor cells often take apoptosis as the desired results. However, mitotic catastrophe and senescence are also promoted by clinically relevant doses of radiation. Furthermore, p53 is a well-known transcription factor that is closely associated with radiosensitivity and radiation-induced cell death. Therefore, we aimed to investigate the involvement of radiosensitivity, cell death modalities and p53 status in response to carbon-ion radiation (CIR) here. Isogenic human colorectal cancer cell lines HCT116 (p53^+/+^ and p53^−/−^) were irradiated with high-LET carbon ions. Cell survival was determined by the standard colony-forming assay. 53BP1 foci were visualized to identify the repair kinetics of DNA double-strand breaks (DSBs). Cellular senescence was measured by SA-β-Gal and Ki67 staining. Mitotic catastrophe was determined with DAPI staining. Comparable radiosensitivities of p53^+/+^ and p53^−^^/−^ HCT116 colorectal cells induced by CIR were demonstrated, as well as persistent 53BP1 foci indicated DNA repair deficiency in both cell lines. Different degree of premature senescence in isogenic HCT116 colorectal cancer cells suggested that CIR-induced premature senescence was more dependent on p21 but not p53. Sustained upregulation of p21 played multifunctional roles in senescence enhancement and apoptosis inhibition in p53^+/+^ cells. p21 inhibition further increased radiosensitivity of p53^+/+^ cells. Complex cell death modalities rather than single cell death were induced in both p53^+/+^ and p53^−^^/−^ cells after 5 Gy CIR. Mitotic catastrophe was predominant in p53^−^^/−^ cells due to inefficient activation of Chk1 and Chk2 phosphorylation in combination with p53 null. Senescence was the major cell death mechanism in p53^+/+^ cells via p21-dependent pathway. Taken together, p21-mediated premature senescence might be used by tumor cells to escape from CIR-induced cytotoxicity, at least for a time.

## Introduction

Colorectal cancer (CRC) is the fifth common malignancy and the fifth leading cause of cancer deaths in China [1]. Radiation therapy is one of the major therapeutic strategies with effective local control and protection of normal tissues for patients with CRC. Still, patients experience metastasis or recurrence after radiation treatment leading to poor prognosis. Usually, studies of therapy or radiation interactions on tumor cells often take the promotion of apoptosis as the desired cell-killing effect [2]. However, the significance of alternative apoptosis-independent therapeutic regimens for cancer treatment caused by mutations and/or deficiencies in the apoptotic signaling pathways have been suggested. Numerous recent studies have revealed that senescence is an important response mechanism to radiotherapy in which cancer cells escape from apoptosis and instead enter into a prolonged cell cycle arrest [3].

Once DNA damage induced by ionizing radiation remains unrepaired, cells driven by DNA damage response (DDR) enter into cell cycle arrest progression, which may maintain a permanent state known as senescence. Persistent radiation-induced foci are considered as a biomarker for cellular senescence and survival risk [4, 5]. Senescence-associated markers also include flat and enlarged morphology, elevated senescence associated-β-galactosidase activity, and the activation of the р53/р21 and p16/Rb pathway.

Senescence has been regarded as an indispensable cellular response for cancer prevention and treatment. Additionally, senescence affects the cellular renewal capacity, suggesting a congruent relationship between the extent of radiation-induced senescence and radiation sensitivity [6]. The growth arrest of senescence is generally thought to be irreversible. One potential reason for the high recurrence of some cancers after low-LET radiotherapy could be the increase in senescent cells that can occur after treatment [7]. Recently, ionizing radiation-induced premature senescence was again linked to p53 function, cells without functional p53 will not undergo a permanent cell cycle arrest and senescence, but will die by other mechanisms, such as necrosis and mitotic catastrophe [8, 9]. However, it is largely unknown the contribution of senescence induced by high-LET ionizing radiation.

There are few reports on the role of cellular senescence in colon cancer cells irradiated by high-LET radiation. One publication reported that carbon-ion beam irradiation effectively kills HCT116 CRC cell lines by predominantly inducing apoptosis of the p53^+/+^ cells and mitotic catastrophe in p53^−/−^ cells rather than the senescence [10]. Instead, proton radiation promotes CRC initiation and progression by inducing senescence-associated inflammatory responses using a human familial adenomatous polyposis syndrome susceptible mouse model [11]. Therefore, it still remains to be clarified whether carbon-ion radiation (CIR)-induced senescence is a potent mechanism for increasing the efficiency of anticancer treatments or a protective mechanism of tumor cells to evade radiation-induced cytotoxicity. A better understanding of the senescent mechanisms could help us to further understand the biologic effectiveness of CIR, which provides insights for the development of more effective, targeted cancer therapies.

## Results

### Cell-killing effects of high-LET CIR were independent of the p53 status

**Fig. 1:**
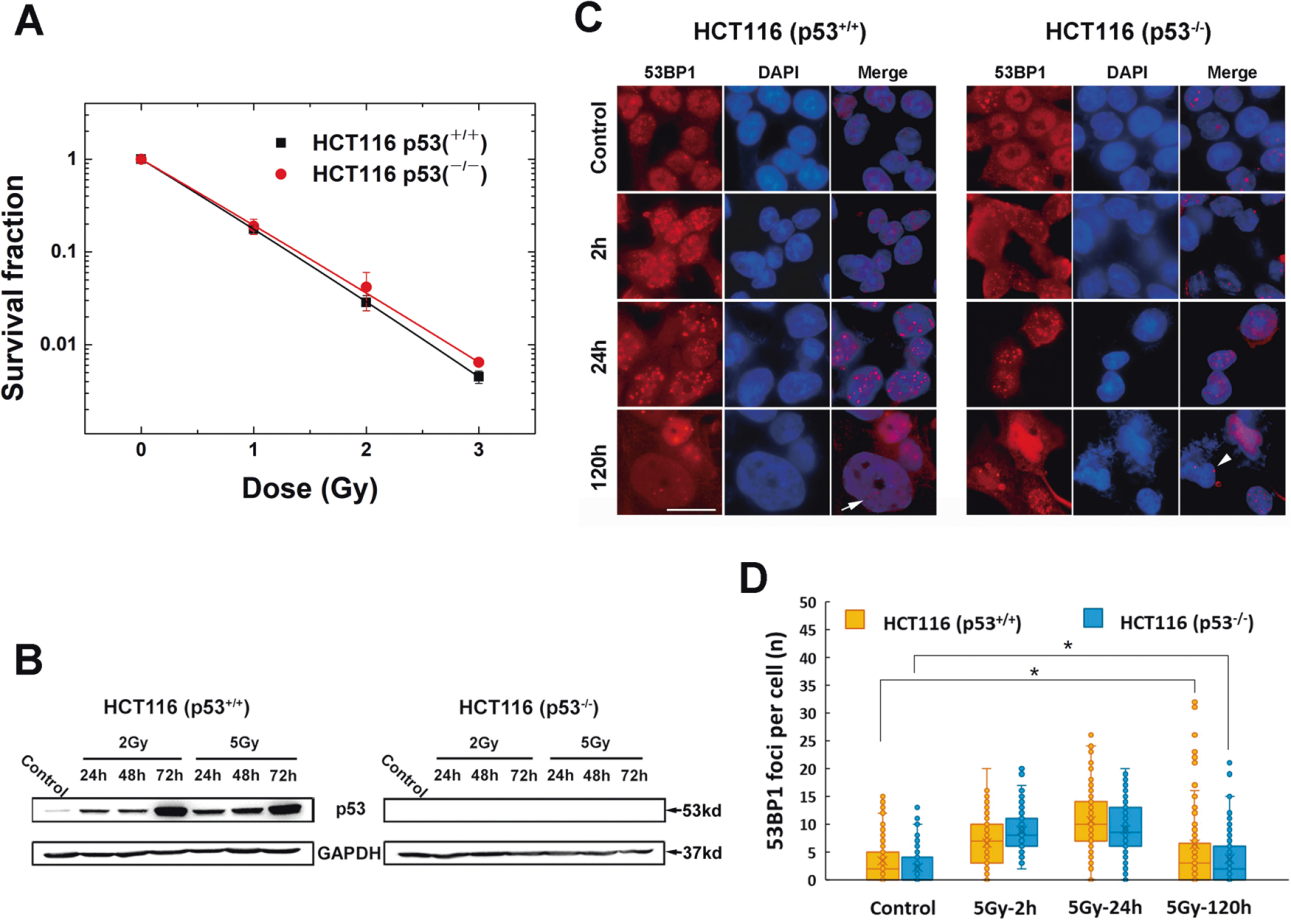
Survival curves of p53^+/+^ and p53^−/−^ HCT116 cells after exposure to CIR (LET = 50 keV/µm) as well as representative images and statistics analysis showing recruitment and retention of 53BP1 induced by 5 Gy CIR. **A** Survival curves for p53^+/+^ and p53^−/−^ HCT116 cells following CIR. Data points represent the mean ± standard error (SE) of three independent experiments. **B** The verification of p53 expression by immunoblot after exposure to CIR. GAPDH level was used as a loading control. **C** Representative images of 53BP1 foci at various time points (2, 24, and 120 h) in p53^+/+^ and p53^−/−^ HCT116 cells. White arrow indicates 53BP1 foci in enlarged and flattened cells; White arrowhead indicates 53BP1 foci observed in multilobulated nuclei. Scale bar: 20 µm. **D** Boxplots showing 53BP1 expression levels in both p53^+/+^ and p53^−/−^ HCT116 cells. A total of at least 50 cells were analysed at each time point from three independent experiments. Boxplots show median, upper and lower quartiles (boxes) and percentiles (whiskers). Data points represent the mean ± standard error (SE) of three independent experiments.

The radiosensitivities of p53^+/+^ and p53^−/−^ HCT116 cells to CIR were measured with the clonogenic survival assay and are shown in Fig. 1A. The survival curves of the two cell lines were close, indicating that their sensitivities to CIR were comparable regardless of the p53 status.

### DNA double-strand breaks generated by high-LET CIR showed irreparable repair kinetics

The numbers of 53BP1 foci formed at 2, 24, and 120 h in both p53^+/+^ and p53^−/−^ cell lines were measured using the immunofluorescence assay after CIR exposure (Fig. 1C). Due to the uneven distribution of foci in cells, median value was used for statistics analysis. In p53^+/+^ cells, 53BP1 foci per cell peaked at 24 h after 5 Gy CIR irradiation. Although it obviously decreased at 120 h, the foci number was still significant compared to the corresponding control (*p* < 0.05, Fig. 1D), indicating the residual unrepairable DNA damages. In p53^−/−^ HCT116 cells, 53BP1 foci per cell kept a constant median level of 8 from 2 to 24 h, till reduced to 2 at 120 h (Fig. 1D). Like p53^+/+^ cells, the foci number per cell also demonstrated irreparable DNA damage in contrast to the control at 120 h (*p* < 0.05, Fig. 1D). 53BP1 foci were also observed in flattened and enlarged p53^+/+^ cells (Fig. 1C, white arrow). In p53^−/−^ cells, 53BP1 foci were observed in the increased population of multilobulated nuclei (Fig. 1C, white arrowhead).

### Expression of cell cycle associated proteins

**Fig. 2:**
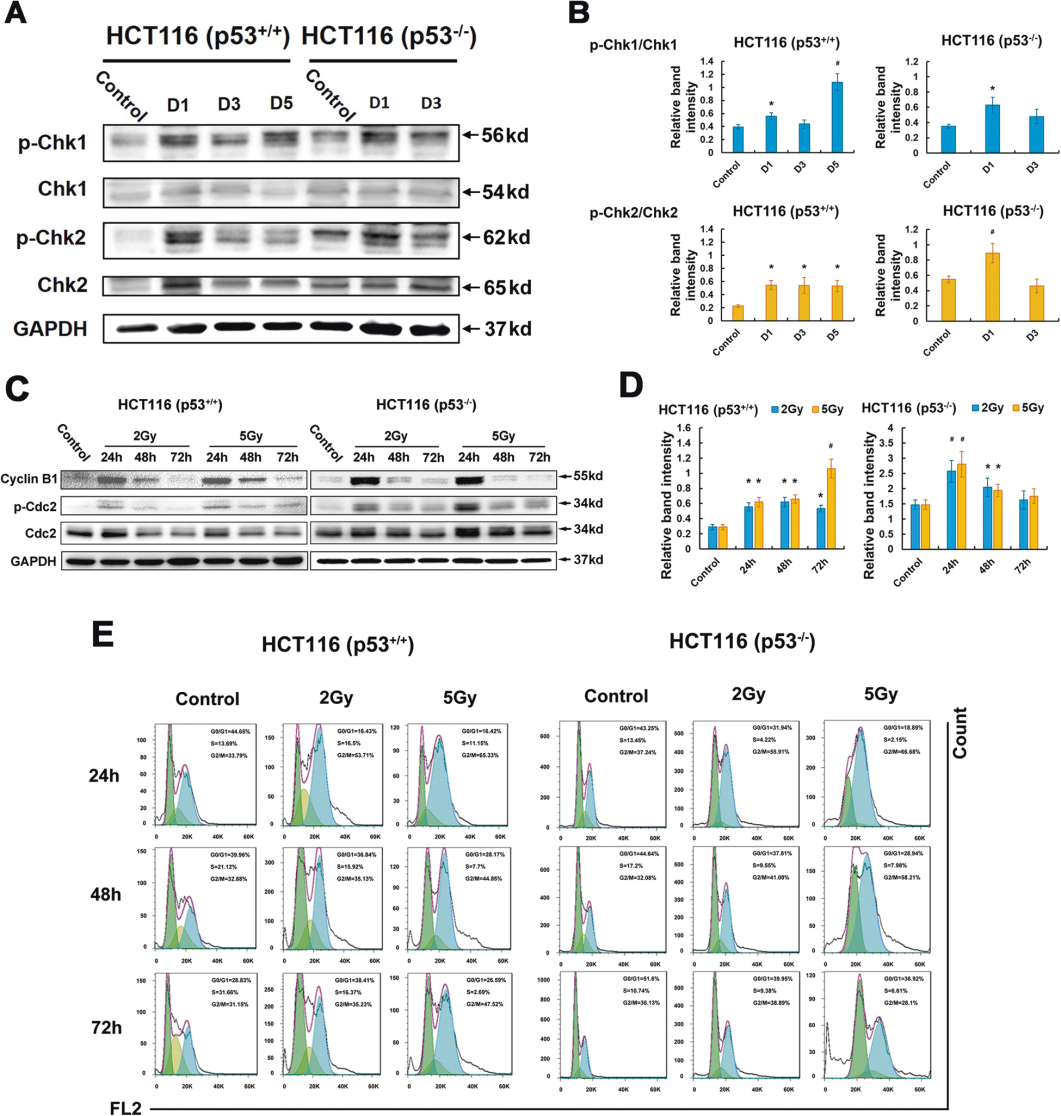
Effect of CIR on the phosphorylation of Chk1, Chk2, and Cdc2 in irradiated p53+/+ and p53^−/−^ cells. **A** The relative band intensities for p-Chk1, Chk1, and for p-Chk2, Chk2. D represents Day. **B** The panel shows the relative band intensities for p-Chk1 and Chk1 and for p-Chk2 and Chk2. Blue columns and yellow columns indicate p-Chk1/Chk1 and p-Chk2/Chk2, respectively. Data are expressed as the mean ± SE. *p < 0.05; ^#^*p* < 0.01. **C** G_2_-M checkpoint alteration in p53^+/+^ and p53^−/−^ HCT116 cells detected by immunoblots of Cyclin B1 and p-Cdc2 following 2 Gy and 5 Gy CIR. **D** The relative band intensities for p-Cdc2 and Cdc2. Data are expressed as the mean ± SE. *p < 0.05; ^#^*p* < 0.01. **E** CIR-induced cell cycle arrest in p53^+/+^ and p53^−/−^ cells for 24, 48, and 72 h using flow cytometry. No less than 72 h prolonged arrest at G_2_-M phase was observed in p53^+/+^ cells.

We analyzed the phosphorylation of Chk1 and Chk2 proteins, two kinases responsible for DNA damage and the G_2_-M checkpoint (Fig. 2). The phosphorylation of Chk1 was increased at day 1 after 5 Gy CIR and persisted for at least 5 days in p53^+/+^ cells. Western blot analyses revealed the maximum increase of phosphorylated Chk1 was at day 1 then decreased in p53^−/−^ cells after 5 Gy CIR. In p53^+/+^ cells, phosphorylation of Chk2 maintained at least for 5 days. While p-Chk2 increased at day 1, then went down to the level less than the control at day 3 in p53^−/−^ cells (Fig. 2A, B).

The inhibition of Cdc2 phosphorylation at Thr161 site can enhance the G_2_-M checkpoint [12]. Cdc2 was phosphorylated at 24 h in p53^+/+^ cells following 2 Gy and 5 Gy CIR, then dephosphorylated, indicating the enhancement of G_2_-M checkpoint in p53^+/+^ cells (Fig. 2C, D). In contrast, p-Cdc2 maintained phosphorylation, although the phosphorylation degree was gradually weakened, demonstrating the impairment of G_2_-M checkpoint in p53^−/−^ cells. Cell cycle measurement performed by flow cytometry also proved the prolonged G_2_-M arrest maintained no less than 72 h in p53^+/+^ cells (Fig. 2E).

Altogether, these results demonstrate that CIR-induced pronounced G_2_-M accumulation in p53^+/+^ cells. In p53^−/−^ cells, transient activation of cell cycle inhibiting proteins indicate impaired G_2_-M checkpoint.

### CIR induces premature senescence in isogenic HCT116 colorectal cancer cells depending on p21

**Fig. 3:**
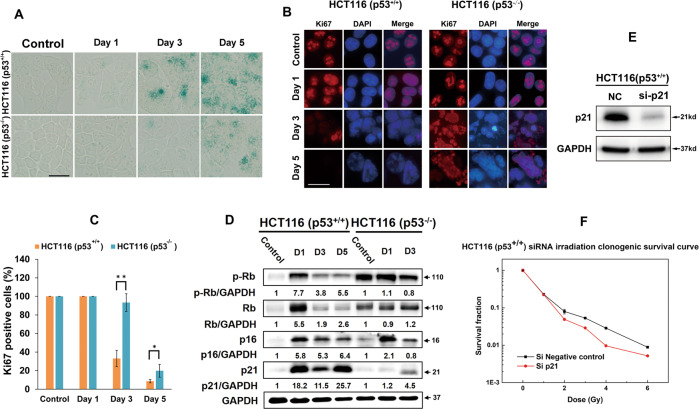
CIR induces premature senescence in isogenic HCT116 colorectal cancer cells depends on p21 but not p53. **A** Senescent cells were determined with β-galactosidase staining at day 1, 3, and 5. Scale bar: 20 µm. **B** Representative images of Ki67 distribution at the indicated time points. Scale bar: 20 µm. **C** Quantification of the mean number of Ki67 positive cells post-irradiation. The values of all experiments represent the mean ± standard error (SE) of three independent experiments performed in triplicate. **D** Senescence pathway activation mediated by kinetics of p21 and p16/pRb expression was measured by western blotting. The intensities of the protein expression bands were quantified against GAPDH from the untreated control cells and are presented as fold increases below the respective lanes. D represents Day. **E** The efficiency of p21 silencing was demonstrated by western blot analysis. NC‚ negative control. **F** Clonogenic survival of p53^+/+^ cells transfected by siRNA p21 (Si p21) or siRNA NC. 24 h after transfection, cells were irradiated at the indicated doses. Data represent the mean of three independent experiments in triplicate, and error bars represent standard error (SE).

G_2_-M arrest launched senescence has been proved by numerous publications [13]. Combined with the indication of persistent 53BP1 foci, senescence was examined using SA-β-gal staining. The results reveal a substantial increase of SA-β-gal positive senescent cells in irradiated p53^+/+^ cells from day 3 following 5 Gy CIR (Fig. 3A). SA-β-gal positive staining was also observed in p53^−/−^ cells, indicating that CIR induces senescence independent of p53. Similar results were also proved by Ki67 staining, a marker of cell proliferation (Fig. 3B). Cell proliferation was abolished from day 3 post-radiation in p53^+/+^ cells upon 5 Gy CIR, in which the Ki67 positive cells accounted for 33.0% and 93.3% in p53^+/+^ and p53^−/−^ cells. Till day 5, Ki67 positive cells dropped to 8.6% and 19.8% in p53^+/+^ and p53^−/−^ cells, respectively (Fig. 3C). Together, these findings demonstrate that CIR induces different degrees of senescence in isogenic HCT116 CRC cells.

To explore the mechanisms underlying the enhancement of cellular senescence by 5 Gy CIR, we evaluated p53/p21 and p16/pRb activation, which are reported to crucially determine the senescence in response to IR [2, 14] (Fig. 3D). p21 was dramatically upregulated and maintained a quite high level for at least 5 days. p16 and pRb were also expressed in the similar pattern as p21. In p53^−/−^ cells, they were only upregulated transiently, then decreased even lower than the level of the control cells at day 3. Clonogenic survival of p53^+/+^ cells transfected by siRNA p21 in combination with CIR indicated that an increase in cell-killing effect when compared to siRNA NC transfection (Fig. 3F). The results suggests that p21-mediated senescence benefits cellular cytotoxicity escape in p53^+/+^ cells induced by CIR, for a time at least.

### Aberration in p53 determined the different mode of cell death upon CIR

**Fig. 4:**
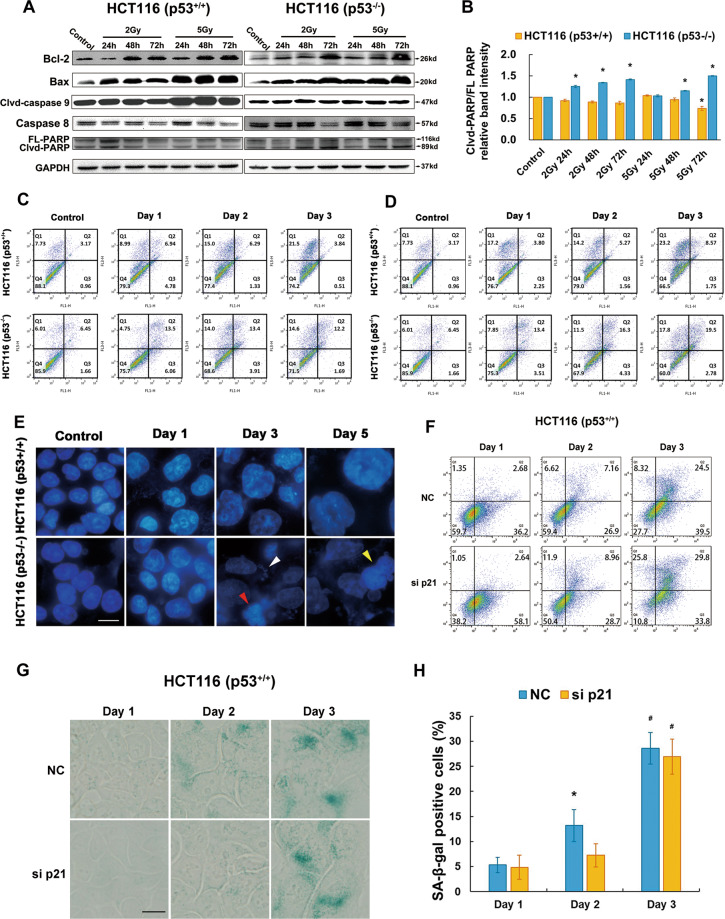
Apoptotic resistance in p53^+/+^ HCT116 cells and mitotic catastrophe prevailing in p53^−/−^ HCT116 cells. **A** Immunoblots to detect key proteins involved in apoptotic pathways, including Bcl-2, Bax, caspase-9, caspase-8 and PARP activation upon 2 Gy and 5 Gy CIR. **B** Quantification of cleaved-PARP (clvd-PARP)/full length PARP (FL-PARP) was performed via densitometric analysis of the western blot technique. Data are expressed as the mean ± SE. *p < 0.05. **C**, **D** Apoptosis was evaluated with Annexin V/PI double staining using FACS at day 1, 2, and 3 in p53^+/+^ and p53^−/−^ cells after 2 Gy (**C**) and 5 Gy (**D**) irradiation. **E** Mitotic catastrophe was measured with DAPI staining at day 1, 3, and 5. Scale bar: 10 µm. **F** Dot plots of p53^+/+^ cells after Annexin V/PI double staining assay. Cells were transfected by NC and p21 siRNA for 48 h then treated by 5 Gy CIR. **G** Senescent cells were determined with β-galactosidase staining. Cells were detected in p21 silencing p53^+/+^ cells compared with NC cells at day 1, day 2, and day 3 after 5 Gy CIR. Sample staining was set at day 1, day 2, and day 3 considering the tendency of cells to fall out when they grow for a long time. Scale bar: 10 µm. **H** Senescent fractions were detected in p21 silencing p53^+/+^ cells compared with NC cells at day 1, day 2, and day 3 after 5 Gy CIR. The percentage is represented as the mean ± SE of three independent experiments. *p < 0.05; ^#^*p* < 0.01.

A growing body of evidence has documented that senescence induction in tumor cells is apoptotic resistance [15]. Therefore, we detected the apoptotic activation following CIR. As an indicator of the apoptotic death (Fig. 4A), no observable elevation of PARP was present in p53^+/+^ cells (Fig. 4B). In contrast, it displayed a delayed increase pattern, reaching highest levels at 72 h following 5 Gy irradiation in p53^−/−^ cells. The apoptotic proportion obtained from flow cytometry also support the above results (Fig. 4C, D). Consequently, CIR does not effectively induce apoptosis in p53^+/+^ cells, especially 5 Gy dosage. It is the reason why we utilized 5 Gy as the irradiation dose to find the potential mechanisms.

Different from p53^+/+^ cells, the proportion of senescent population in p53^−/−^ cells was relatively low. Following 5 Gy irradiation, vast micronuclei (white arrowhead), multilobulated (red arrowhead) and multinucleated (yellow arrowhead) nuclei were increased with time, which are the characteristic phenotypes of mitotic catastrophe (Fig. 4E).

To further prove p21 is capable of inhibiting apoptotic cell death and promoted cellular senescence, Annexin V/PI double staining assay and SA-β-Gal staining in p53^+/+^ cells were performed after transfection by siRNA p21 in combination with 5 Gy CIR. The results show that early apoptotic cells transfected by siRNA p21 were significantly more than those transfected by siRNA NC (58.1 versus 36.2) (Fig. 4F). This difference disappeared at day 2. Till day 3, there was no obvious difference between the two groups of cells. After p21 silence, the fraction of senescent cells at day 2 were obviously less than that in NC group. Other than that, no significant differences were found between the two groups (Fig. 4G, H). Therefore, p21 silencing is involved in a decreased senescence and an increased apoptotic rate in p53^+/+^ cells following 5 Gy irradiation.

**Fig. 5:**
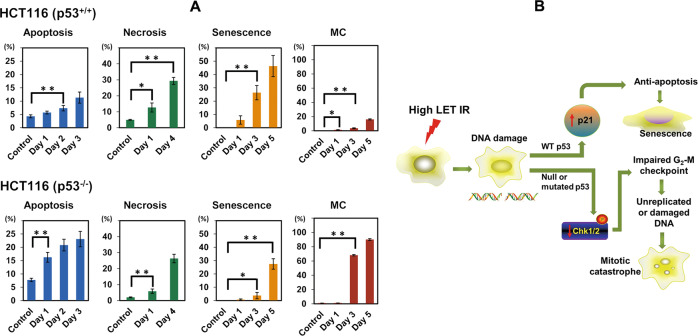
Statistical analysis of cell death modalities in p53^+/+^ and p53^−/−^ cells following 5 Gy CIR and schematic illustration of predominant cell death modalities in isogenic HCT116 cells after CIR. **A** Data are presented as the mean ± standard error (SE) of three independent experiments. *p < 0.05; **p < 0.01. Note that the percentages in the ordinate are shown in different scales for the different death modes. MC represents mitotic catastrophe. **B** Schematic illustration to describe the predominant cell death mechanism of isogenic HCT116 cells after CIR.

To ascertain the modes of cell death induced by 5 Gy CIR in irradiated p53^+/+^ and p53^−/−^ cells, the necrosis was also detected. Complex cell death modalities rather than single cell death were observed in both p53^+/+^ and p53^−/−^ cells after 5 Gy CIR (Fig. 5A). Among them, senescence was found to be the major cell death mechanism in p53^+/+^ cells while mitotic catastrophe was the main cell death mode in p53^−/−^ cells. In p53^−/−^ cells, cells positive for mitotic catastrophe reached to 90% at day 5 post-irradiation, which might explain why we could not harvest enough proteins at day 5 in p53^−/−^ cells (Fig. 3D).

## Discussion

Biological effectiveness of CIR has been investigated for decades. The results obtained up to now substantiate that CIR is more effective than X-rays in killing tumor cells [16]. Nevertheless, only a few studies have addressed the mechanisms of cell death involved in response to CIR, most of which focused on apoptosis only [17, 18]. As to apoptosis, the results so far have reached a general consensus that apoptosis induced by high-LET radiation is not affected by cellular p53 gene status [17, 19, 20]. We have noted that some publications considered that apoptosis and the genes controlling it, like p53, played little or no role in the sensitivity of cancer cells to anticancer drugs and radiation, when clonogenic survival was used to assess cell-killing effects [21]. Actually, several studies have suggested that chromatin structure could be the main modulator that influences radiosensitivity, one of the mechanisms is to alter susceptibility to death processes [22]. Therefore, apoptosis and p53 may not determine cellular radiosensitivity, but it could reflect physiological response to specific treatments, which further guide us to design effective therapeutic regimens on the basis of physiological and biochemical events including but not limited to death modalities. In the present study, apoptosis is not the exclusive or even primary mechanism of cell death induced by CIR.

Complex DNA damage induced by high-LET CIR, also referred to as clustered damage, is more difficult to repair compared with simple DNA damage, resulting in serious biological consequences, one of which is cell death. Cumulative irreparable DSB lesions may have a causal role in triggering cellular senescence [23]. In the present study, persistent 53BP1 foci, as a biomarker for DNA repair deficiency, were observed till 120 h after CIR in both cell lines. As a result, senescence and mitotic catastrophe, as main cell death modes, were respectively observed in p53^+/+^ and p53^−/−^ cells after 5 Gy CIR.

It has been established for a decade that p21 is capable of suppressing apoptosis at different levels. Among them, one of the mechanisms is that p21 prevents cells from apoptosis by suppression of the CDK (Cyclin-dependent kinase) activity that seems to be essential for activation of caspase-9 and caspase-3 downstream of the mitochondria after 20 Gy γ-ray irradiation [24]. Sustained upregulation of p21 is also crucial for the maintenance of the stress-induced premature senescence program consequent to therapeutic exposures [15, 24–27]. Although the observations from the studies above were obtained from high doses of low-LET radiation or chemicals, our data provide important evidence to prove the observations of PARP inactivation and apoptosis inhibition, concomitantly with the predominant cellular senescence in p53^+/+^ cells after high-LET CIR radiation. Undoubtedly, sustained overexpression of p21 plays a multifunctional role in inhibiting apoptosis upon CIR as well as promoting cell senescence. Utilization of siRNA p21 indicated an increase in cell-killing effect of CIR, suggesting that the induction of senescence might be used by tumor cells to escape from radiation-induced cytotoxicity.

In p53^−/−^ cells, our results demonstrated that mitotic catastrophe was predominantly induced by 5 Gy CIR. Chk1 and Chk2 are key signal transducers in the DNA damage checkpoint signaling pathway [28]. Especially, the phosphorylation of Chk1 at Ser345 has a critical role in activation and maintenance of the G_2_-M checkpoint [29–31]. Coupled with the important role of p53 for DNA repair, and accordingly, mitotic catastrophe was predominantly induced in p53^−/−^ cells.

## Materials and methods

### Cell lines

HCT116 CRC cells with wild-type p53 and their isogenic derivatives that lack p53 (HCT116 p53^−/−^) were generous gifts of Dr. Bert Vogelstein (Johns Hopkins University, Baltimore, MD). The cells were maintained in DMEM medium (Gibco) containing 10% fetal bovine serum (FBS, Corning), 100 U/mL penicillin, and 100 μg/mL streptomycin (Gibco).

### Irradiation

Some cells were irradiated with a carbon-ion beam accelerated by the Heavy Ion Medical Accelerator in Chiba (HIMAC) at the National Institute of Radiological Sciences (NIRS), Japan, which provides a horizontal beam line for biological sample irradiation. The initial energy of the carbon-ion beam was 290 MeV/u. Some of the irradiations were performed with a carbon-ion beam of 165 MeV/u in the heavy ion therapy terminal of the Heavy Ion Research Facility in Lanzhou (HIRFL) at the Institute of Modern Physics (IMP), Chinese Academy of Sciences. The LET value of both the beams was adjusted to be ~50 keV/μm when traversing the cell samples. Doses of 0, 1, 2, 3, and 5 Gy were applied in this study. Independent triplicate experiments were performed. All samples were irradiated at room temperature.

### Clonogenic assay

Cell survival was determined by the standard colony-forming assay. Briefly, cells were replated at a density of about 100 surviving cells into 60 mm Petri dishes supplemented with DMEM medium including 10% fetal calf serum after irradiation. After incubation for 14 days, the cells were fixed and stained with methyl blue solution. Colonies with more than 50 cells were counted as survivors.

### Immunofluorescence assay

53BP1 and Ki67 were visualized to identify double-strand breaks (DSBs) and cell proliferation. After irradiation, cells were fixed with 4% paraformaldehyde in PBS at room temperature and subsequently immunostained for corresponding detection. Briefly, the fixed cells were permeabilized with 0.5% Triton-X-100 and subsequently blocked with 10% goat blocking serum for 60 min. Monoclonal primary antibody (Abcam, Cambridge, MA) was used at a dilution of 1:200 in 1% (m/v) BSA in PBS and incubated at room temperature for 2 h. The slides were further incubated with TRITC-conjugated secondary antibody (1:2000 dilution) for 60 min at room temperature. Nuclei were counterstained with mounting medium with DAPI (1.5 µg/ml, VECTASHIELD Mounting Medium, Vector Lab, Inc., United States). Coverslips were mounted to slides and viewed using a BX51 fluorescent microscope (Olympus, Tokyo, Japan). At least 50 cells should be counted per image.

### RNA interference

p21-targeting siRNA (#1026) was purchased from BIONEER (Korea). 24 h before transfection, plate 6 × 10^5^ cells in each well without antibiotics. Dilute siRNA duplex and Lipofectamine RNAiMAX (#13778150, ThermoFisher Scientific) in Opti-MEM^®^ (#31985-070, ThermoFisher Scientific). Incubate this solution 5 min at room temperature. Mix the diluted siRNA duplex with the diluted Lipofectamine RNAiMAX and incubate for 20 min at room temperature. Add the mixture to each well containing cells. Incubate the cells for 5–6 h at 37 °C in CO_2_ incubator. Change fresh medium containing serum and incubate till the time point to collect the samples.

### Western blotting

After removal of the media, treated and untreated cells were rinsed twice with ice-cold PBS, and the samples were lysed with RIPA buffer (Beyotime Inc., NanTong, China) supplemented with a protease and phosphatase inhibitor cocktail (Roche, Basel, Switzerland). The lysates were collected by scraping from the flask and then centrifuged at 13,000 rpm at 4 °C for 5 min. Proteins (30 μg) were loaded on 10% or 12% SDS-polyacrylamide gels for electrophoresis, transferred onto polyvinylidene difluoride (PVDF) membranes (Millipore, Boston, USA), blotted, and probed using specific primary antibodies and the corresponding secondary antibodies. Caspase-8 (#9746), caspase-9 (#9501), p53 (#9282), Bax (#5023), PARP (#9542), phosphorylated Chk1 (Ser345, p-Chk1, #2348), phosphorylated Chk2 (p-Chk2, #2197), phosphorylated Rb (p-Rb, #8516) and phosphorylated Cdc2 (p-Cdc2, #9114) antibodies were purchased from Cell Signaling Technology (MA, USA). GAPDH (60004-1-Ig), p16 (10883-1-AP), Bcl-2 (60178-1-Ig), Bax (50599-2-Ig), p21 (10355-1-AP), Chk1 (60277-1-Ig), Chk2 (13954-1-AP), Cyclin B1 (55004-1-AP), and Cdc2 (67575-Ig) antibodies were from Proteintech (Wuhan, China). Anti-Rb (#554136) antibody was from BD Biosciences (Franklin Lake, USA).

### SA-β-Gal staining

HCT116 p53^+/+^ and HCT116 p53^−/−^ cells (respective 1 × 10^5^ cells) were plated in 35 mm cell culture dishes and incubated for 24 h before exposure. At each indicated time point after irradiation, cells were stained with the Senescence Associated β-Galactosidase Staining Kit (C0602, Beyotime, China) following the standard protocol suggested by the manufacturer. Senescent cells were identified under a light microscope.

### Apoptosis detection

After treatments, cells were washed with PBS and resuspended in 400 μL binding buffer. The cell density was adjusted to 1 × 10^6^. Then cells were counterstained with 5 μL Annexin V-FITC (60 μg/mL) and 10 μl PI (40 μg/mL) solution and incubated for 15 min in the dark at room temperature. Ten thousand events were collected and analyzed on a flow cytometer cell sorter (BD FACS-Calibur, USA).

### Necrosis detection

Cells were quantified with a double staining of Hoechst 33342 and PI detection kit according to the manufacturer’s instructions. Detection using flow cytometry cell sorter was conducted at different time points. Necrotic cells were obtained by counting the percentage of Hoechst 33342^+^/PI^+^ cells.

### Statistical analysis

Statistical analysis was performed using the Student’s *t* test. Data were reported as mean ± standard error (SE). Differences with a *p* value < 0.05 were considered statistically significant. Protein expression levels were quantified with the ImageJ software (v4.6.2). Survival curves were plotted with the Origin 9.0 software while the other figures were plotted with Excel 2019 software.
